# Supplementary figures and images for: Adaptation prevents the extinction of Chlamydomonas reinhardtii under toxic beryllium
Source: PeerJ. 2016 Mar 21;4:e1823. doi: 10.7717/peerj.1823 (PMC4806628; doi:10.7717/peerj.1823)

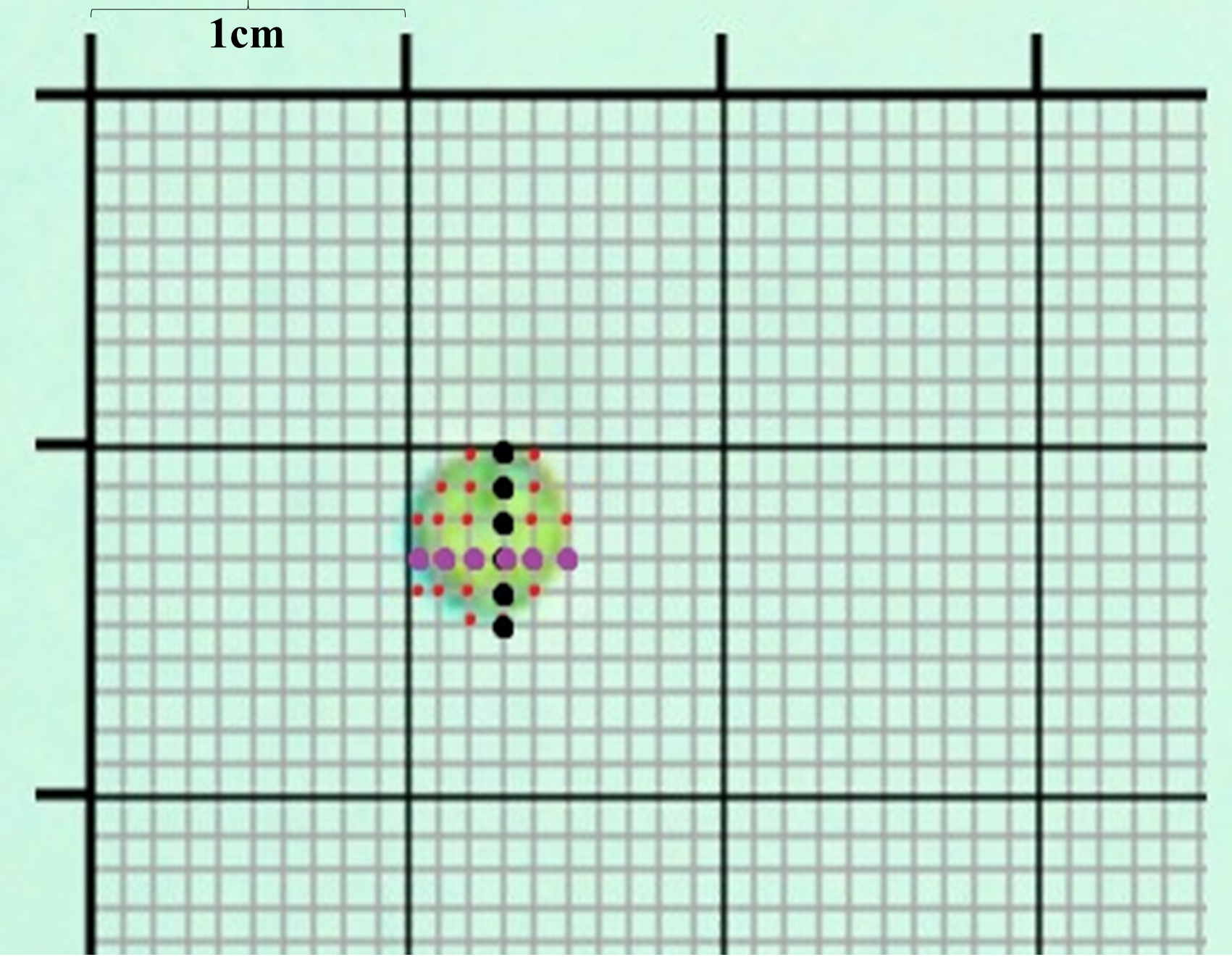

Supplement: Figure S1 — This figure represents the point-counting procedure based on planimetry techniques for morphometric analyses. Measurements were made on optic microscopy and ultrathin TEM photographs taken at 12000× of both sensible and resistant cells to beryllium. Data collection was carried out with a 1 mm precision squared template. Morphometric analysis requires the use of 3 variables: larger diameter of cells illustrated with black points (d); lower diameter (D) (purple points) and finally the number of apexes which including the cells, giving an estimation of cell volume. [file peerj-04-1823-s001.png]

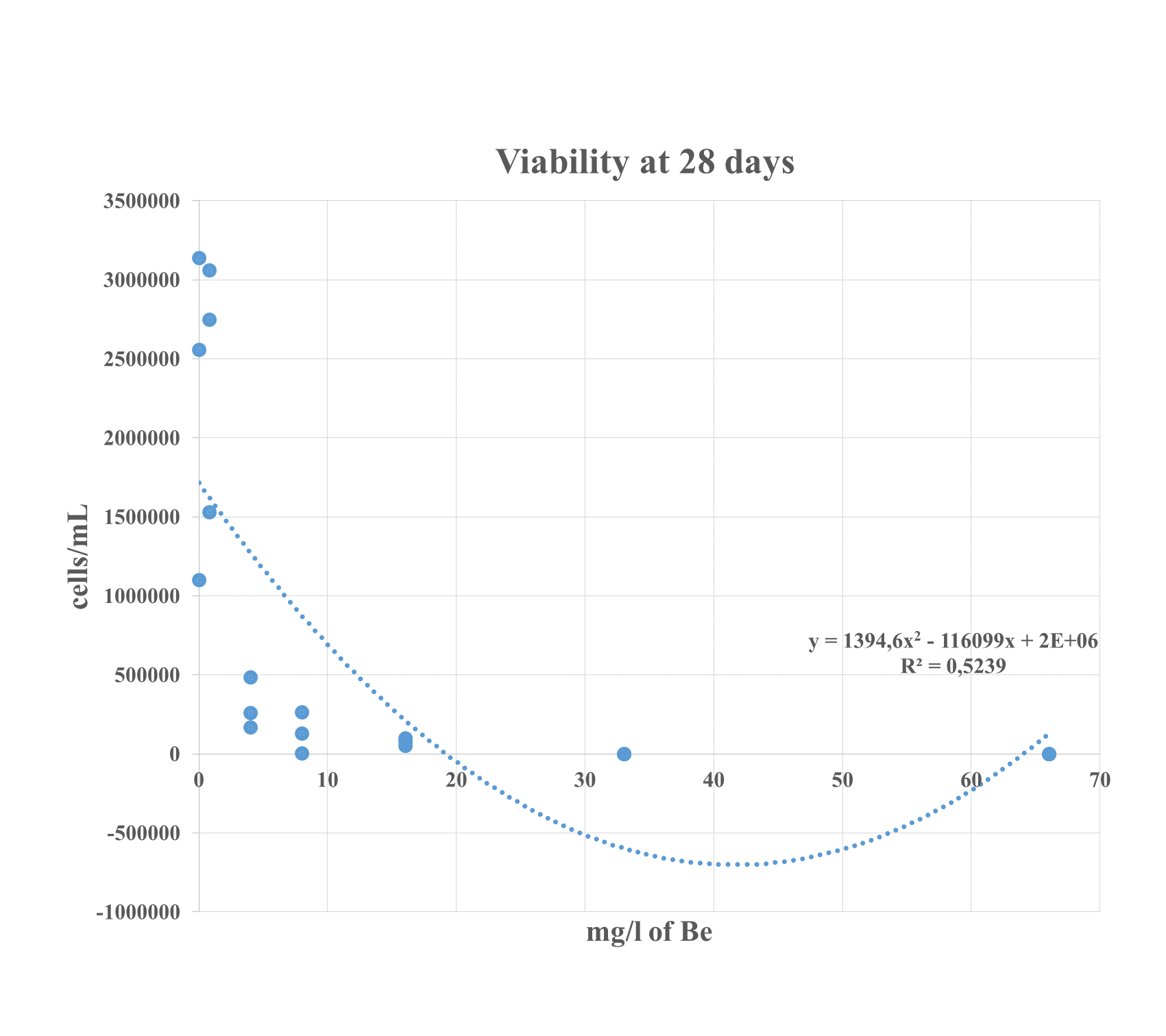

Supplement: Figure S2 — This figure represents the polynomial regression of the wild-type Chlamydomonas sp. viability (represented as number of living cells per mL) exposed to increasing doses of beryllium (from non-exposed to 33 mg/l) at 28 days. The points represent the values of each of the replicates per dose. [file peerj-04-1823-s002.png]

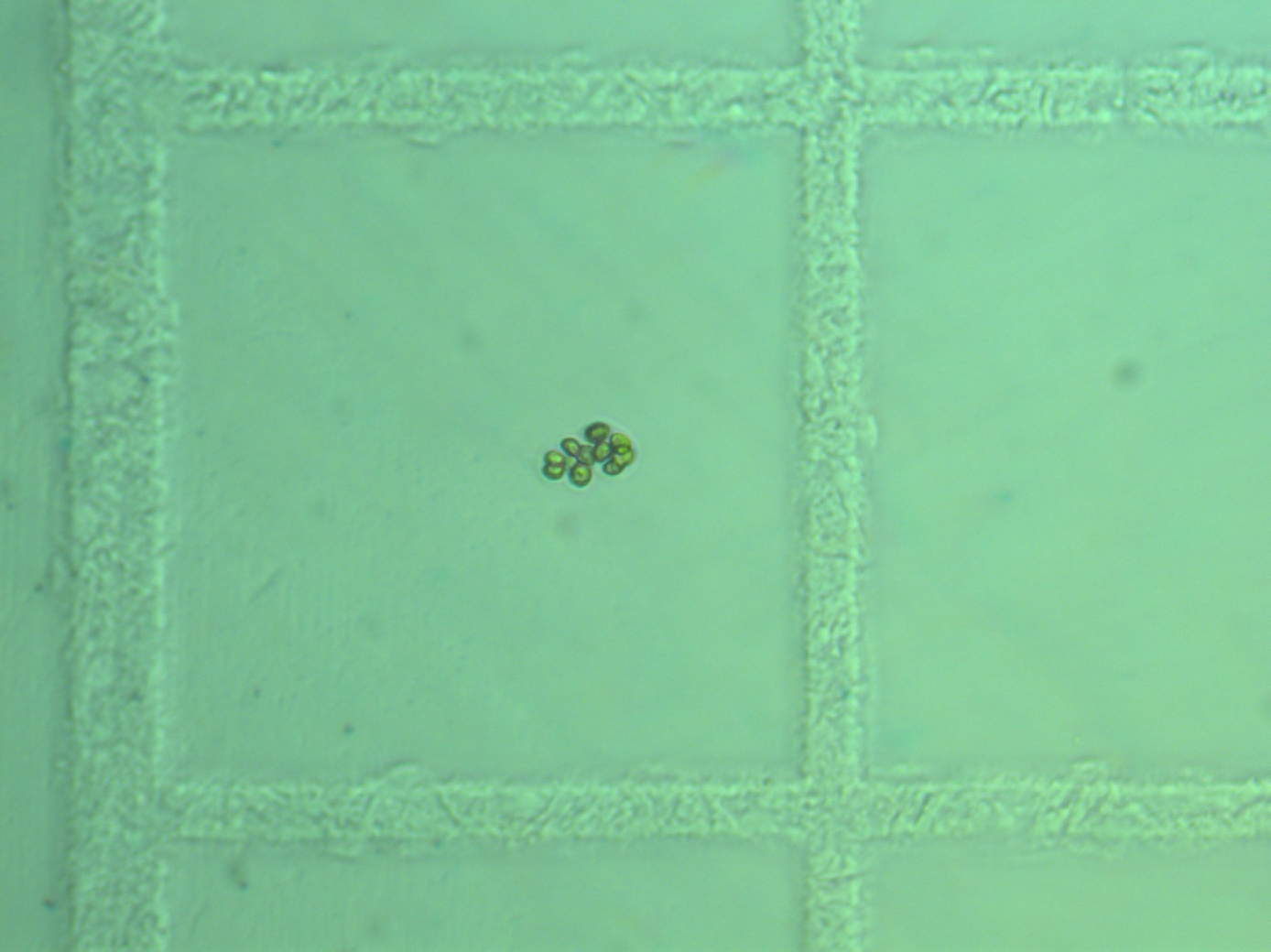

Supplement: Figure S3 — Images were taken in an optic microscope with Uriglass shelter chambers. The strain behavior under selective conditions suggest the formation of palmelloid colonies. [file peerj-04-1823-s003.jpg]
